# Hospital and Institutional News

**Published:** 1918-07-20

**Authors:** 


### HOSPITAL AND INSTITUTIONAL NEWS.

### THE MILITARY HOSPITAL INSPECTORS.

Until the King's Fund was established, the weakness of the voluntary system lay in the absence of any inspection, because the hospitals were (and still are) mostly too self-centred to take a wide view and form a general standard, while only one gentleman was found whose back was broad enough to bear the smacks of the offended institutions. These considerations do not weigh with the Army, and the *British Medical Journal* affirms that inspectors of military hospitals have been appointed to all the Commands of England, except to those of London and Aldershot. The Inspector of Hospitals in the Northern Command is Major-General Bruce Skinner, C.B., C.M.G.; of the Southern, Sir T. P. Woodhouse, K.C.M.G.; of the Eastern, Sir R. H. Whitehead, K.C.B.; and of the Western, Major-General R. Porter, C.B. All the inspectors, we understand, have had considerable previous experience with the armies in the field.

### A GREAT STEP FORWARD AT BLACKBURN.

Thanks to the friendly co-operation of Mr. Nathan Smith, the superintendent of the Blackburn and East Lancashire Infirmary, we are able to record the steps by which a scheme of workpeoples' contributions has been accepted in principle, with a hint, too, of an attempt to organise later on a wider plan of sectional subscriptions. As The Hospital has previously noted, financial difficulties showed no sign of being met, and in March last a meeting of delegates from representative trades throughout the area considered a scheme of weekly contributions. The delegates adjourned to consult their respective associations, and at their reunion last week a promising resolution was passed, the text of which is given in Mr. Nathan Smith's informing letter on page 354. Mr. Laurence Cotton, the Mayor of Blackburn, has lent invaluable aid. Over a hundred delegates met on the earlier occasion, and among them were the Mayors of Accrington and of Darwen. Sixty years ago, under the then Mayor, workers' representatives agreed to support the infirmary. But since 1907, notwithstanding the growth of Blackburn, their contributions have hardly grown at all. The success at Preston and Birmingham was explained by men with personal knowledge of how it was attained. Previous extensions have been mainly paid for by the wealthy, and the need for improvements is great. The kitchen itself is over fifty years old, and an eye, ear, nose, and throat department, an $x$-ray unit, a venereal diseases clinic, and a bacteriological department are all necessary. The absence of debt gives a clear start.

### THE WIDER ISSUE.

Therefore, now that a special committee of infirmary representatives of the nurses' home, and of each trade union has been appointed, the scheme should quickly get to work. But in applauding this beginning let us seek a richer inspiration in the future which the new scheme opens up. One step must be taken at a time. The effort must be persistent, not sporadic. This granted, let us note with pleasure that the wider issue was raised by the chief objector to the resolution. Why, asked Mr. T. Smith, the president of the Weavers' Association, in urging a delay which the meeting wisely would not tolerate, why should the workers be appealed to alone? He suggested, for instance, that the shopkeepers and licensed victuallers should be invited to give similar organised help. Mr. Smith is right. In due course, when the present scheme is working smoothly, all its supporters should understand that it is the first step in the new policy: the organisation of sectional subscriptions from every class which the hospital serves. This policy alone can meet the problem of the future which is to maintain the new departments and the added beds which are in part necessary now, and will be imposed by circumstances after the war. If the workers do their share and set an example which is followed, the wealthy can again be relied on to provide the cost of the new buildings required.

### A BIG SCHEME AT CARDIFF.

Details are now available of the scheme by which the authorities of King Edward VII.'s Hospital and the Lord Mayor of Cardiff intend to raise £34,000 so that the standing disgrace of a waiting-list of 11,000 names may be removed by the provision of 200 further beds for their accommodation. There are reckoned to be 150,000 workers in the district. If 140,000 will subscribe or collect 5s. a head, £34,000 is at once secured. Each collector or subscriber of 5s. is a recruit, and each friend of the hospital, like the enthusiasts of the College of Nursing, is asked to " find " a company, a battalion, even a brigade, according to the measure of their spirits and capacity. The district is mapped out. Each centre, the name of which is published, is asked to raise a definite quota which is based upon the number of 5s. recruits which may be reasonably expected from the population of the area. A scheme of this size on this principle has never, we think, been so deliberately undertaken before, and Cardiff, which in the hospital sense is the home of victorious causes, will no doubt be true to its tradition of success.

### FEMINISTS AND THE SOUTH LONDON HOSPITAL.

The anniversary celebrations at the South London Hospital for Women will have taken place after we have gone to press this week, but the three principal means by which money is to be raised should prove collectively successful. Purses and gifts will be received by the Duchess of Sutherland at the hospital on the south side of Clapham Common. A sale of useful articles presided over by the Mayoress of Wandsworth and Mrs. Harry Greer, wife of the new member for Clapham, will follow, and a concert will finish the afternoon. Outside a street collection will take place in Clapham, Balham,

Tooting, and Streatham. Tickets for the function at the hospital cost one shilling, and (we emphasise the good news) include tea. They are mostly obtainable at the hospital and in Clapham, Balham, and at 235 Lavender Hill, from which we infer that an effort is being made to appeal to those districts from which the hospital patients are chiefly drawn. A hospital for women nowadays, especially one staffed by women, is a popular type of institution. We are all feminists now! Therefore it is well to remind enthusiasts that, this popularity notwithstanding, these hospitals find it difficult not merely to secure a modest endowment to pay their capital expenses on building and so on, but to offer salaries to their staffs equal to those which are now the rule in hospitals staffed by men. This fact shows that the "cause" is not yet wholly victorious, and those who espouse it have still to secure that wider measure of support which is the proof of complete success.

### THE DAY OF RECKONING AT THE GUEST HOSPITAL.

A hospital which is short of money is not necessarily unpopular, but it is no use for an unpopular hospital to issue an appeal. The latter seems to be the unfortunate position of the Guest Hospital, Dudley, where many passive protests against the administration and the method of appointment to the staff have culminated in the fiasco which threatens the recent appeal for £6,000. Even the local newspapers, which are usually timidity itself in publishing opinions or facts of common local knowledge, lay the blame for the failure of the appeal at the Guest Hospital's own doors. Quite rightly, in the circumstances, they contrast the £30,000 raised in one day in Birmingham for the Children's Hospital with the £6,000 which in many days cannot be found in Dudley. They remark that the chairman has declared the hospital to be "a private enterprise," a close concern. They pillory the administration as "archaic." They call for a review of the old deeds which were drawn up in days when the hospital was dependent upon *private* bounty. They urge the claim of the subscribers to be listened to—as well as heard. They speak of the "gag" at the chairman's elbow, and the "many unredressed complaints." They point to the "obsolete policy" which has ended in depriving the hospital alike of public confidence and public money. That this is no idle criticism can be seen from the fact with which the *Dudley Herald* drives home its argument, the fact that the beds have been cut down from 100 to fifty. This is the familiar proof of incompetence. On this fact alone we are prepared to believe that there is "an old gang" and a close committee with a few official henchmen who should be cleared out. In strangling the hospital the policy of the committee has already recoiled upon itself, and it should confess its ineptitude and retire.

### THE LAST LAP OF LEEDS EXTENSION.

The Lord Mayor of Leeds recently launched a great appeal for £100,000 on behalf of the Leeds General Infirmary. There is uncertainty in respect to the objects in view, which are three—first, to replace £50,000 worth of securities which have had to be sold during the war; secondly, to supply the remaining £25,000 needed to complete the sum required for the King Edward Memorial Extension; lastly, to provide a reserve of £25,000 out of which the exceptional expenses incurred through war conditions may be met. Nearly a third of the whole sum has been received or promised. In support of the scheme, the Lord Mayor remarked that not only was Leeds as famous for its hospitals as for its commerce, but the original estimate for the bold extension policy had been closely adhered to. Since the war the expenses had jumped up by one-third, and the necessary sale of securities which ensued had reduced the permanent income by one-third. The three new wards raised the cost of maintenance by £9,000 a year, and since the war 1,000 more in-patients had been treated. The Workpeople's Hospital Fund Committee was so well satisfied with the results that it proposed to double, if possible, its already big subscription. This is the best justification of the bold policy of the past and of the new financial programme. Those who recall the condition of the infirmary at the time when The Hospital published its independent report have cause for thankfulness that the changes then advocated have been carried out. The report, in fact, was the beginning of the new era which, under the guidance of Mr. Charles Lupton, has increased the reputation and public usefulness of the General Infirmary immensely.

### LORD DURHAM'S CHARGE AGAINST THE RED CROSS.

At a meeting in aid of the Durham Light Infantry Prisoners of War Fund, the Earl of Durham severely criticised the British Red Cross Society. He felt very sore, he said, at the Red Cross claim that it was feeding all prisoners of war. The claim, besides being inaccurate, discouraged subscriptions to local funds. He knew what the Durham Fund had done. If the Red Cross boasted that it was the only barrier between our prisoners of war and starvation, why had the Society not sent subsidies to the Durham County Association? The County of Durham prisoners would not starve whatever the Red Cross did or did not, and that was why he appealed so earnestly for the local fund. Rather than have any patronage from the Red Cross, good heavens! if he had any money he would pay the bill himself. He hoped the people of the North would not allow themselves to be under the thumbs of the Central Committee, which he distinctly charged with claiming more than it had accomplished. It was announced during the meeting that Lord Durham had promised to find £200 a month, so that the sincerity of his local patriotism has (incidentally) been vindicated.

### SCABIES FROM STRAP-HANGING.

Our contemporary *Truth* has a correspondent who has something alarming to say about the strap which, in these times of over-crowded vehicles, is so essential to the safety of the many who have to travel standing. He announces that the Local Government Board are about to issue advice and suggestions for dealing with the extraordinary prevalence of scabies throughout the country, which is, he says, largely a war-effect imported by soldiers on leave. He thinks it may interest Londoners especially to hear, on the authority of an eminent practitioner, that one of the most fruitful causes of infection of this unpleasant condition is the now customary "strap-hanging" in the carriages of the underground railways and in the trams and 'buses of the metropolis. The favourite temporary home of the parasite (*Acarus scabiei*) is between the fingers of the hand, and it does not require a very vivid imagination to discern that the straps in the vehicles mentioned are a most convenient medium for the transfer of the parasite from one passenger to another. It is therefore to be hoped that one of the instructions will be the periodical removal and disinfection of the straps, which, instead of being permanently fixed overhead, as at present, ought to be so hung as to facilitate this simple operation.

### TOMMIES AND TOWN PLANNING.

DR. MARION PHILLIPS, the writer on social questions, was the lecturer at the Manor Gardens Annexe of the Great Northern Central Hospital on the last Friday in June, when she chose "Housing" for her subject. She pointed out that while building had been falling behind before the war it has now practically ceased, so that the shortage has become a famine, and a reasonable relief of the dreadful overcrowding, bad enough before and almost intolerable now, can hardly be looked for till three or four years after the war ceases. Such relief cannot be brought about unless the problem is tackled both by the Government and by the local authorities. The scarcity of building material will make Government control a necessity. After a few more remarks on the general question of providing a sufficient number of houses, Dr. Phillips dealt with the peculiar defects of the great majority of working-class houses to-day—the crowding together, the absence of space at the back, the bad lighting, the inconvenient sinks and ranges. She might have discussed the evil of privies the only opening of which is on to the stairs. Town planning and the possibility of reform depend as much on "land" as on any other factor. The interest taken by the men was evidenced by the questions they asked the lecturer, whom they invited to address them next session.

### EXCESS PROFITS AND HOSPITAL SUBSCRIPTIONS.

IN a speech in support of the great appeal for the Leeds General Infirmary, which we discuss elsewhere, Mr. Charles Lupton, the distinguished chairman, raised a very important matter. Firms which he had already approached for subscriptions asked that an effort be made to have their subscriptions charged against their working expenses. If his urgent appeal to the authorities had been successful, the effect would have been to charge subscriptions to hospitals against working expenses before the question of excess profits was discussed. A similar arrangement was already in force in regard to the clubs for munition workers, and he had told the authorities that the provision of hospital advantages for the working classes was even more important than a supply of social clubs. He took the matter to the Ministry of Munitions, and from there to the Treasury, to be met at last with the reply that the law would need alteration before the suggestion could be carried out. His point, in short, could not be conceded without a new Act of Parliament. The matter has been officially shelved for the moment. But we must not be discouraged. Hospitals have done so much for the country and the Government during the war that it would be a small and deserved return to place them on the same footing as the social clubs in respect to the heading under which the subscriptions of firms should be allotted.

### MORE HOSPITALS FOR AMERICANS.

To provide further hospital accommodation in this country for wounded Americans the Army Council has arranged to take over the Southern and North-Eastern Hospitals of the Metropolitan Asylums Board, and the Portsmouth Corporation has agreed to hand over the local mental hospital.

### WHERE STATISTICS WOULD BE USEFUL.

IT would be interesting to know the maximum time that new patients are kept waiting at casualty departments of the various hospitals before being seen by a doctor. It is no uncommon thing to hear patients complain bitterly of the long hours which they are compelled to wait. A hospital exists for the sake of the sick poor, and it should be an understood fact that no poor sick person is detained unnecessarily. How long an interval should elapse between the visits of the casualty officer? Why should not statistics be kept of the hours of waiting in each case, If they were, and published as part of the annual report, we believe that the number of "unavoidable" delays would be reduced, to everyone's advantage.

### A REMARKABLE INQUIRY AT BERMONDSEY.

MR. J. S. OXLEY, Local Government Board inspector, is conducting a public inquiry into an extensive purchase of drapery goods for the institutions of the Bermondsey Board of Guardians. In the months of May and June last year Mr. E. Pitts Fenton, the clerk, acting under a resolution adopted by the Guardians, and confirmed by the Local Government Board, empowering him in conjunction with Mr. W. H. Ecroyd, J.P., L.C.C., the chairman of the Contracts Committee, to purchase supplies without advertising for tenders, bought drapery goods to the value of £22,321. It is contended that the purchases were far in excess of the requirements of the institutions, and were more than sufficient, in many cases, to cover the demands for ten and more years. These figures, however, are based on a pre-war consumption, and although at the present time the number of the indoor poor belonging to Bermondsey has considerably decreased, this is counterbalanced by the number of boarders sent into the Bermondsey institutions from elsewhere. But a far greater reason for the large purchases of drapery is that the Ladywell Institution belonging to the Bermondsey Guardians has been taken over by the War Council as a military hospital, and Mr. Fenton maintains that the certified accommodation for wounded soldiers has been raised to 1,760. The Guardians have to provide drapery and bed-linen for the soldiers, who require more linen than the ordinary patient. It is anticipated that the inquiry will last a considrable period.

### DRUGS FORBIDDEN TO MEMBERS OF HIS MAJESTY'S FORCES.

An Army Council Order, published in the *London Gazette*, notifies additional restrictions on the sale of drugs to members of His Majesty's Forces. To such members, except doctors, dentists, and veterinary surgeons, no person is to sell the following drugs: barbitone, benzamine lactate benzamine hydrochloride, chloral hydrate, coca, cocaine, codeine, diamorphine, Indian hemp, opium, morphine, and sulphonal and its homologues, and any salts, preparations, derivatives, or admixtures prepared from or with any of these drugs, unless a written prescription is produced, signed by a registered medical practitioner. The prescription must be marked, "Not to be repeated," and the drug must not be supplied more than once on the same prescription. Chemists must be careful to retain the prescription, as it must be kept open to inspection by any person authorised by a Secretary of State.

### SMALL HOSPITALS AND THE INCREASED DUTY ON ALCOHOL.

Whilst the large increase in the duty recently imposed on alcohol has made rectified spirits of wine much dearer, this extra duty may be reclaimed if it can be shown that such spirits have been used to make "medicinal preparations." In a previous Budget the principle was recognised that medicinal preparations were worthy of exemption from excessive duty, and whilst the larger hospitals have reclaimed the extra duty, in many cases to the extent of several hundred pounds, some of the smaller institutions have found that the clerical work involved in making the necessary return has been out of proportion to the amount saved, and they have therefore not made a claim for repayment of duty. Now, however, that the duty is so heavy it would appear necessary for every small hospital, if it values economy, to make a reclaim for the duty. The necessary forms of declaration required may be obtained from the Local Supervisor of Customs for the district concerned. The alternative is to buy all spirituous medicinal preparations ready made, and, in this case, the wholesale druggist makes the claim for rebate, fixing the price charged on this basis. It should be noted that flavouring spirit like peppermint or lemon is not classed as a medicinal preparation for which a claim for repayment of the additional duty may be made.

### SHOULD UNQUALIFIED DISPENSING BE FORBIDDEN BY LAW?

Mr. A. H. Jenkin, chairman of the Public Pharmacists' Association, raised the question of the dispensing of medicines by unqualified persons at a recent meeting of the Pharmaceutical Council. It appears there is no law to prevent anyone from dispensing medicines—even those containing potent and poisonous preparations—and that, unless the compounded prescription is sold, *i.e.*, a money payment made for it, no action may be taken at law against the dispenser. To remedy this state of affairs, Mr. Jenkin proposes that the matter be referred to the special committee set up by the Pharmaceutical Society to consider the Ministry of Health proposals, with the view of collecting evidence on the subject and making a report to the Council. In so far as this question affects the interests of hospital pharmacists they will, no doubt, welcome the action taken by Mr. Jenkin. But to what extent is dispensing by private persons practised? One has heard of a peer who dabbled in chemistry as a hobby, but we recall no case of danger resulting therefrom.

### THIS WEEK'S DRUG MARKET.

Business continues quiet, and price changes are not, on the whole, of particular note. So many weeks have passed since the time when practically all the changes were in an upward direction that one is inclined to believe the general upward tendency has received a check. Certainly fluctuations in an upward direction are fewer than they were, and a downward tendency is noticeable in the prices of several drugs. The market needs careful watching, and the prudent buyer will proceed cautiously. In view of the shipping difficulties bulky drugs which come from overseas are almost bound to be dearer. The demand for quinine is quieter, but the value is firmly maintained, and is likely to be for the present in view of the smallness of the stocks. Business has been done in aniseed oil at higher rates; American peppermint oil also tends upwards in price, and the same may be said of bergamot oil and clove oil. Hypophosphites are rather dearer. On the other hand, several articles have a rather lower price tendency, as, for instance, artificial oil of wintergreen, hexamine, acetanilide, salol, phenacetin, and paraldehyde. The value of imported saccharin is firmly maintained, and sugar of milk is scarcer than ever.

